# A Novel Utility Function for Energy-Efficient Power Control Game in Cognitive Radio Networks

**DOI:** 10.1371/journal.pone.0135137

**Published:** 2015-08-10

**Authors:** Yousef Ali Al-Gumaei, Kamarul Ariffin Noordin, Ahmed Wasif Reza, Kaharudin Dimyati

**Affiliations:** 1 Department of Electrical Engineering, Faculty of Engineering, University of Malaya, Kuala Lumpur, Malaysia; 2 Department of Electrical and Electronics Engineering, Faculty of Engineering, National Defence University of Malaysia, Kuala Lumpur, Malaysia; University of Reading, UNITED KINGDOM

## Abstract

Spectrum scarcity is a major challenge in wireless communications systems requiring efficient usage and utilization. Cognitive radio network (CRN) is found as a promising technique to solve this problem of spectrum scarcity. It allows licensed and unlicensed users to share the same licensed spectrum band. Interference resulting from cognitive radios (CRs) has undesirable effects on quality of service (QoS) of both licensed and unlicensed systems where it causes degradation in received signal-to-noise ratio (SIR) of users. Power control is one of the most important techniques that can be used to mitigate interference and guarantee QoS in both systems. In this paper, we develop a new approach of a distributed power control for CRN based on utility and pricing. QoS of CR user is presented as a utility function via pricing and a distributed power control as a non-cooperative game in which users maximize their net utility (utility-price). We define the price as a real function of transmit power to increase pricing charge of the farthest CR users. We prove that the power control game proposed in this study has Nash Equilibrium as well as it is unique. The obtained results show that the proposed power control algorithm based on a new utility function has a significant reduction in transmit power consumption and high improvement in speed of convergence.

## Introduction

Growth of wireless communication users and services in recent years causes scarcity in radio spectrum resources. Spectrum measurements indicate that more than 70% of licensed spectrum is underutilized in some locations and times while on the other hand, unlicensed spectrum has been growing dramatically [1]. Cognitive radio (CR) is an empowering technique that has an ability to solve the spectrum scarcity and improve spectrum efficiency. CR can observe the wireless environment, learn the usage pattern of spectrum frequency, and adjust to changing the wireless environment conditions. The effective process to utilize the spectrum is by allowing CRs to access the unused parts of licensed spectrums (spectrum holes) that resulted because of the absence of licensed users. Spectrum sharing between licensed users, and CRs led to an efficient utilization of spectrum, but the presence of CRs causes an increase in interference. Increasing interference causes degradation in quality of service (QoS) of licensed (primary) system that owns the rights over these bands. Therefore, Federal Communications Commission (FCC) proposed an interference temperature metric referred to as “interference temperature” limit in order to limit CR interference and protect primary and CR systems [2].

To mitigate interference produced from CRs during spectrum sharing, an efficient power control algorithm is necessary in CR devices. In addition, power control is essential to maintain the required signal-to-interference-ratio (SIR), and extend battery life of CR devices. The concept of economic and game theory has been used in recent years as an alternative approach to power control problem in wireless data networks, in which service preference was dependent on utility function. The challenge in game theory approach is the formulation of a utility function that it has a physical meaning and the game outcome is not trivial [3]. There are various and different ways to design a power control algorithm based on utility and price functions. Some researchers proposed utility as a difference between utility and pricing functions, and users seek to maximize this utility in a selfish manner. In this case, the utility function should be quasi-concave and an optimal point is selected to be somewhere within the practical parameter range, such as minimum and maximum power, and it depends on other users’ behavior [3]. The special case of this type of utility function is energy efficiency, in which the utility has a physical meaning of the number of successfully received information bits per joule of energy cost. All users adjust their transmit power to achieve the required SIR that is not defined directly inside the utility function, but depends on the supposed efficiency function.

On the other hand, some other researchers proposed the utility (cost) function as a difference between price and utility functions, and users seek to minimize this cost function in a selfish manner. In this case, the cost function should be quasi-convex and the optimal point (minimum point) is selected to be somewhere within the practical parameter. In the cost function approach, all users try to achieve the required SIR (target SIR *γ*_*i*_ ≅ Γ_*i*_) defined directly in the cost function. The differences between the two approaches can be summarized, as shown in Table 1. In the following, we review the most important works on power control based on different utility and cost functions and innovative pricing techniques used for system improvement.

**Table 1:** Differences between utility and cost function based power control.

	Power control based on Utility function	Power control based on Cost function
Definition	Difference between utility and price (Utility-Price)	Difference between utility and price (Price-Utility)
Users goal	Maximize their own utility	Minimize their own cost
Function shape	Quasi-concave (maxima)	Quasi-convex (minima)
Final SIR	The SIR achieved depends on efficiency function that is defined in utility function	The SIR achieved is equal to the target that is declared in cost function
Others	Similarly to optimization theory	Similarly to control theory

In [4][5], authors introduced the utility function that reflects user’s preference regarding to SIR and transmitter power. The utility function in [6] has been defined as the number of information bits received successfully per joule of energy expended, and authors introduced linear pricing combined with the utility function to obtain Pareto improvement to improve system performance. On the other hand, the power control problem has been studied in [7] using game theory based on cost function, in which each user tried to minimize its own cost to achieve the target SIR. The cost function in [7] has been defined as a weighted sum of power and square of SIR error.

The main goal of power control algorithms in cognitive radio network (CRN) context is to maximize spectrum utilization by allowing many CRs to share licensed users. During the existence of CRs (spectrum sharing), all CRs terminals should be working under the interference temperature limit to protect the QoS of primary system as well as the CR system. To achieve this goal, some researchers focus on the development of power control algorithms in the low priority unlicensed network (CRN) and neglect the interaction of high priority network (primary system). In this case, the development of the power control algorithm is similar to those applied in wireless data networks without any effect on the structure of CRN.

Authors in [8] proposed another power control game based on cost function where the target of transmit power has been included as the target SIR. The cost function in [8] is defined as a weighted sum of logarithm of SIR error and logarithm of power error. The algorithm has many advantages, such as fast convergence, better anti-noise, and capacity. Also, the author [9] proposed a non-cooperative power control algorithm based on a cost function similar to [8] by using the square root function instead of logarithm function to speed up their proposed algorithm. Authors in [10] proposed power control game in CRNs based on the cost function and they used two SIR thresholds in order to adjust the interference factor of power and improve the fairness. In our previous work [11], we proposed a new SIR-based sigmoid power control game in CRNs based on the cost function. The cost function was defined as a weighted sum of square of SIR error and a sigmoid weighting factor of power, which resulted on a considerable saving on, transmit power compared to well-known algorithms. More detailed explanations for the concept of game theory and its application in CRNs can be found in [12]. In [13], the authors introduced a certain degree of hierarchy in the non-cooperative power control game to improve individual energy efficiency of all users. The pricing issue of uplink power control game has been studied in [14], in which CRs adjust their transmission power levels to maximize their own utilities; conversely, primary service will charge them on their transmitted power level to enhance its own income. Authors in [15] considered a non-cooperative power control game for maximum energy efficiency with a fairness constraint on the maximum received power of CRs, in which the results indicate that CRs have a beneficial impact on the whole network throughput. Channel and power allocation have been proposed and evaluated in [16] using game theory based on local information, which analyzes the problem under the interference model. The no-regret learning algorithm has been also used in [16] to overcome the convergence limitations of local game and perform joint channel and power allocation.

Utility function via pricing has been also considered in recent years to solve the problem of power control in CRNs. Authors in [17] introduced a concept of target SIR to modify the utility function and also Shuffled Frog Leaping Algorithm (SFLA) to improve the accuracy of the solution. In [18], the power control game has been improved based on the outage probability of primary user in a spectrum-underlay CRN. In addition, authors in [18] designed an efficient swarm intelligent algorithm to improve the convergence speed and improve the energy-efficiency. A payment-based power control scheme based on the non-cooperative power control game has been considered in [19], in which the distance of CRs and the value of SIR were used as a reference for punishment price setting.

In this paper, we proposed a novel utility function that consists of a weighted exponential of the ratio of target SIR and the desired signal, and the pricing function that comprises a power function of CR’s transmitting power. Important features of the proposed power control scheme are: (i) it can preserve the required QoS of all CRs efficiently with insignificant reduction in SIR, (ii) the algorithm can be practically implemented in a distributive manner without requiring additional information, (iii) a significant reduction, and better power allocation to all CRs, and (iv) fast convergence to the Nash Equilibrium. The novelty of the proposed power control scheme is the new sigmoid exponential efficiency function and the power function that applies to the pricing part. The choice of the proposed utility and pricing functions is key to enable each CR to choose its transmitting power efficiently. It guides closest CRs to the base station to achieve their QoS requirement with low cost, whereas it guides farthest CRs from the base station to achieve their QoS requirement with high cost to mitigate the interference. Furthermore, we proved the existence and Nash Equilibrium of the power control game and the conditions of the selected pricing factors. Furthermore, we explained the difference between the linear and power functions to the pricing function, and the effect of weighting factor to the utility function and transmit power. On the other hand, the proposed efficient non-cooperative power control algorithm (EF-NPGP) can be practically implemented in a distributive manner without requiring additional information.

The rest of this paper is as follows. Section 2 describes the system model of CR, the non-cooperative power control game based on the utility function and the EF-NPGP algorithm. Section 3 presents the numerical results and discussion. The limitation of this study and future works are discussed in Section 4. The conclusion is presented in Section 5.

## Materials and Methods

### System model

**Fig 1:**
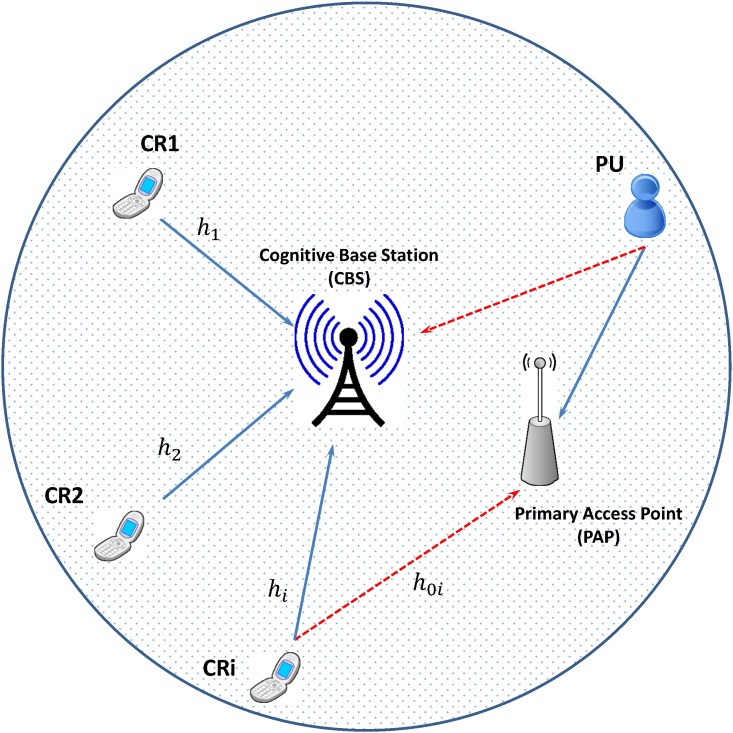
System model of cognitive radio network.

In this paper, we consider a single cell CRN with one cognitive base station (CBS) and one primary access point (PAP) as shown in Fig 1, and focus on uplink power control case. There are *N* CRs sharing the same licensed spectrum with a single primary user and they employ code division multiple access (CDMA) technique to utilize the available spectrum in their own communications. It is assumed that CRs are stationary and distributed inside the coverage area of the cell. We denote *p*_*i*_ to the transmit power of *i*th cognitive user and *h*_*i*_ to the channel link gain (path gain) between the *i*th cognitive user and CBS.

A general formula for SIR in the single cell cognitive radio CDMA system of *i*th CR can be expressed as

$$\gamma_{i}(p_{i})=\frac{G\;p_{i}h_{i}}{\sum_{j\neq i}^{N}p_{j}h_{j}+\sigma_{i}^{2}}\;\geq \;\Gamma_{i}\;,i=1,2,\ldots ,n$$ (1)

where *G* denotes the processing gain of the spread spectrum system, Γ_*i*_ is the threshold SIR, and $\sigma_{i}^{2}$ is the power of the Gaussian noise. The sum of interference, including noise in the denominator of Eq (1) can be denoted as *I*_*i*_(**p**_−*i*_), hence Eq (1) can be rewritten as a function of user transmit power and the transmit power of other users as:

$$\gamma_{i}(p_{i},p_{-\text{i}})=\frac{G\;p_{i}h_{i}}{I_{i}\left(p_{-i}\right)}=\frac{G\;p_{i}h_{i}}{\sum_{j\neq i}^{N}p_{j}h_{j}+\sigma_{i}^{2}}$$ (2)

The subscript −*i* indicates the interference that depends on the power of all users except the *i*th user.

In this model, the primary system objective is to maximize its own revenue (profit) by allowing many CRs to share its own spectrum. The revenue maximizing is restricted by the limited performance degradation of primary users or the interference temperature limit [3].

The total interference power made by CRs should be less than a given threshold that is called interference temperature limit and this constraint can be expressed as

$$\sum_{i=1}^{N}p_{i}h_{0i}\;\leq \;I_{TL}$$ (3)

where *h*_0*i*_ is the channel gain from the transmitter of cognitive radio *i* to the access point of the primary system, and *I*_*TL*_ is the interference temperature limit.

### Non-cooperative power control game with pricing

In recent years, the concepts of microeconomics and game theory have been used to define users QoS in terms of utility (cost) function instead of SIR [3]. In general, the power control game model consists of three elements (i) mobile users (or CRs) that represent the players or decision makers of the game, (ii) power strategy which represents the game strategy or action space, and (iii) utility function (preference of users). Each user in the network seeks to maximize its own utility in a selfish manner. The non-cooperative power control (NPG) game model can be expressed as

$$\Phi =[N,\{P_{i}\},\{U_{i}(.)\}]$$ (4)

where *N* = {1,2,…, *n*} is the index set of players (CRs), $P_{i}=[0,P_{i}^{\text{max}}]$ represents the transmission power strategy set of user *i*, and $P_{i}^{\text{max}}$ is the maximum transmission power of user *i*. The utility function of user *i* is referred to as *U*_*i*_(.), in which each user in the network seeks to maximize its own utility in a selfish manner. To reduce power consumed by CRs, achieve the required SIR, and mitigate interference in CRN; the payoff function (utility function) of the power control game in Eq (4) should consider the following properties as in [20]:

1. The utility is a function of CR’s transmit power and SIR. The SIR of CR is a function of CR’s transmit power and the transmit power of other users.
2. When CR user increases its power level, this will increase its own SIR, but will decrease the SIR of other CRs.
3. For a fixed SIR, the CR prefers the lower power level to higher ones to extend battery life and reduce interference.
4. For a fixed power, the CR prefers higher SIR to lower SIR in order to obtain a good channel condition.

In CRN, similar to any wireless network, each CR transmits its information over the air using multiple access system. Since air is a common medium for all the signals, each CR’s signal acts as interference to other user’s signal. This interference plus fading, multipath and background noise cause signal distortion as its traveling from the source to destination. The denominator of the SIR in Eq (1) represents all these impediments of the signal. In addition, CR users are battery-based devices, so the transmitter power is another important commodity for them. Therefore, SIR and transmit power are the most important parameters that will be used to formulate the expression, which determines user satisfaction using the network [21].

Information sent from transmitters to receivers in wireless data and CRNs are in the form of frames (or packets) of length *M* bits, containing *L* < *M* information bits at a data rate of *R* bits/sec. Assuming that all errors in the received signal can be detected by the system and the incorrect data can be retransmitted. Then, the achieved throughput *T* can be defined as

T=Rf(γ) (5)

where *f*(*γ*) is the efficiency function of transmission. The efficiency function *f*(*γ*) should depend on SIR achieved over the channel, and its value varies from zero to one (i.e., *f*(*γ*) ∈ [0,1]). Further, if the user’s *i* transmitted power is *p*_*i*_, then the utility function of user *i* can be expressed as the number of information bits received successfully per joule of energy consumed as [22]

$$U_{i}(p_{i},p_{-\text{i}})=\frac{L\;R\;f(\gamma_{i})}{M\;p_{i}}$$ (6)

The Nash Equilibrium resulting from non-cooperative power control is inefficient because it ignores the cost (harm) it imposes on other terminals by the interference it generates. Therefore, the concept of pricing has been used to encourage the users to use the network resource more efficiently. The general expression of non-cooperative power control game with pricing (NPGP) can be written as

$$\Phi^{C}=[N,\{P_{i}\},\{U_{i}{}^{C}(.)\}]$$ (7)

where $U_{i}^{\;C}(.)$ is the utility function via pricing that can be defined as

$$U_{i}^{C}(p_{i},p_{-\text{i}})=U_{i}(p_{i},p_{-\text{i}})-C_{i}(p_{i},p_{-\text{i}})$$ (8)

Several works considered the problem of power control by introducing dissimilar utility and pricing functions. In [6], the authors proposed the utility function as

$$\text{NPGP}\;:\;U_{i}^{C}\;(p_{i},p_{-\text{i}})=\frac{L\;R}{M\;p_{i}}{\left(1-e^{-\gamma_{i}/2}\right)}^{M}-C_{1}\;p_{i}$$ (9)

where *C*_1_ is the positive pricing factor. Based on Eq (9), authors in [17] used the same utility function in [6], and they introduced a new pricing function where the non-cooperative power control game (NPG) was established using the modified Shuffled Frog Leaping Algorithm (MSFLA) as follows

$$\text{NPG}-\text{MSFLA}\;:\;U_{i}^{C}\;(p_{i},p_{-\text{i}})=\frac{L\;R}{M\;p_{i}}{\left(1-e^{-\gamma_{i}/2}\right)}^{M}-C_{2}\;e^{p_{i}}-C_{3}\;\left(\gamma_{i}-\Gamma_{i}\right)$$ (10)

where *C*_2_ and *C*_3_ are positive pricing factors. The efficiency function that has been used in [6] and [17], is the same, which is related to the non-coherent frequency shift keying (FSK) modulation scheme. The efficiency function is expressed as

$$f_{1}(\gamma_{i})={\left(1-e^{-\gamma_{i}/2}\right)}^{M}$$ (11)

In [18], the authors proposed a utility function depending on the sigmoid function, and they introduced a new designed pricing function as defined where the non-cooperative power game with pricing (NPGP) was established using the efficient swarm intelligent algorithm (ESIA) as

$$\text{NPGP}-\text{ESIA}\;:\;U_{i}^{C}\;(p_{i},p_{-\text{i}})=\frac{L\;R}{M\;p_{i}}\frac{1-e^{-\gamma_{i}}}{1+e^{\Gamma_{i}-\gamma_{i}}}-\alpha \;e^{\beta ((\gamma_{i}/\Gamma_{i})-1)}\frac{p_{i}}{p^{th}}$$ (12)

where *α* and *β* are the positive pricing factors, *p* ^*th*^ is the average interference power which can be obtained by taking the mean value of $p_{i}^{th}\;:\;p^{th}=\left(p_{1}^{th}+p_{2}^{th}+\ldots .+p_{i}^{th}\right)/N$, and the sigmoid efficiency function is expressed as

$$f_{2}(\gamma_{i})=\frac{1-e^{-\gamma_{i}}}{1+e^{\Gamma_{i}-\gamma_{i}}}$$ (13)

The fair power control game in [19] proposed the utility function based on the simplified sigmoid function used in [18], and they introduced a new non-linear pricing function where the non-cooperative power game with pricing (NPGP) was established using a sliding model, called (R-NPGP) as

$$\text{R}-\text{NPGP}\;:\;U_{i}^{C}\;(p_{i},p_{-\text{i}})=\frac{L\;R}{M\;p_{i}}\frac{1}{1+e^{\Gamma_{i}-\gamma_{i}}}-\mu \;\lambda_{i}\;\frac{p_{i}}{p^{th}}$$ (14)

where *μ* is a positive pricing factor and *λ*_*i*_ is another pricing factor that varies for different CRs based on their generated conditions, and the efficiency function is expressed as

$$f_{3}(\gamma_{i})=\frac{1}{1+e^{\Gamma_{i}-\gamma_{i}}}$$ (15)

### Proposed Game model

**Fig 2:**
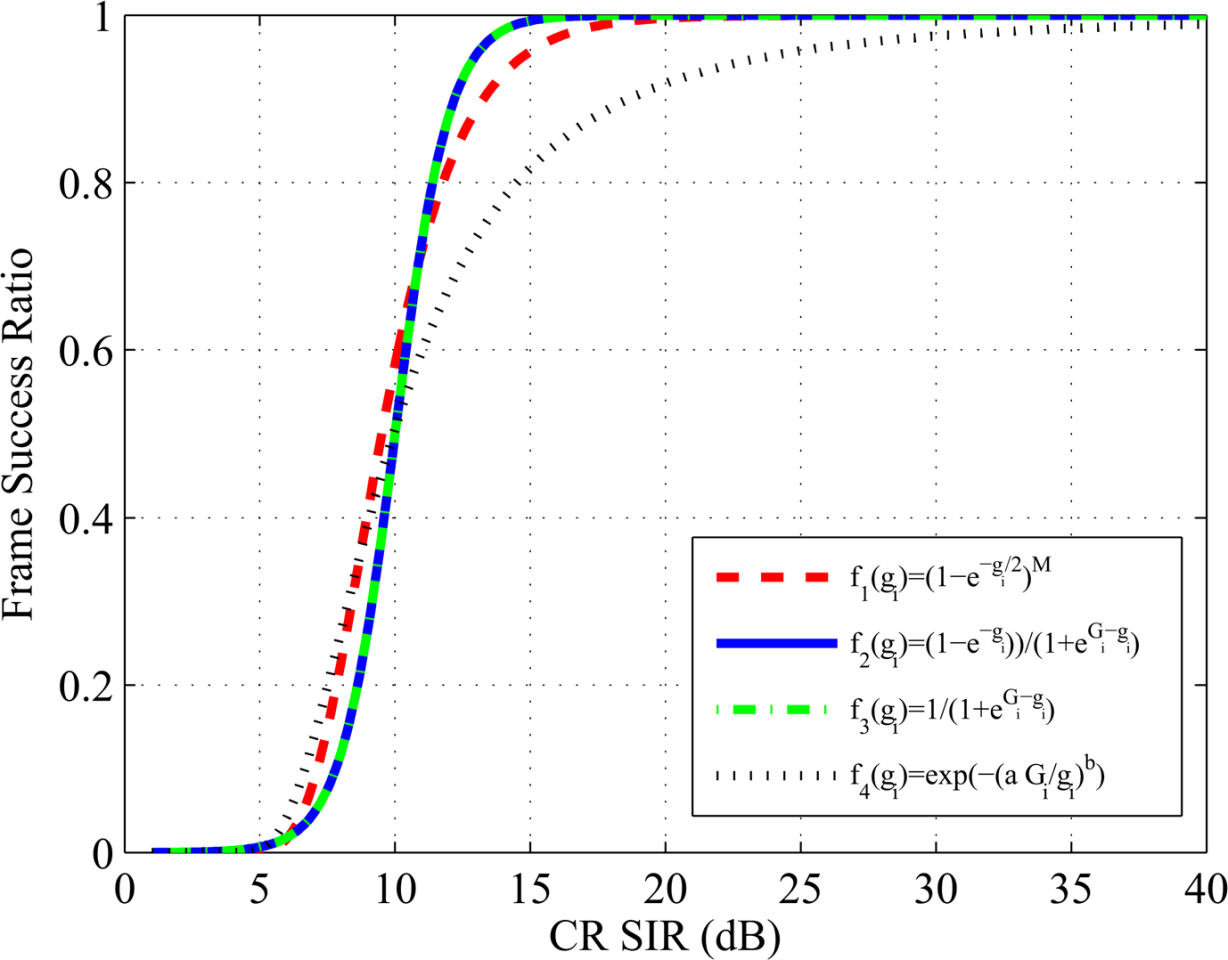
Efficiency function comparison Γ_*i*_ = 10, *a* = 0.8, *b* = 3, *M* = 80.

In this subsection, we propose a novel utility function based on a new sigmoid efficiency function and a power function of user’s transmit power pricing function. We introduce a sigmoid efficiency function as the exponential ratio of target SIR and the desired signal as

$$f_{4}(\gamma_{i})=\text{exp}\left(-{\left(\frac{a\;\Gamma_{i}}{\gamma_{i}}\right)}^{b}\right)$$ (16)

where *a* and *b* are non-negative weighting factors. The efficiency function in Eq (16) is a sigmoidal function with *f*(∞) = 1, and *f*(0) = 0 to ensure *U*_*i*_ = 0 when *p*_*i*_ = 0. Moreover, the efficiency function *f*_4_(*γ*_*i*_) with data rate *R* represents the throughput of the system. The comparison of the above efficiency functions is shown in Fig 2. According to Eq (6), the utility function of the *i*th CR can be written as

$$U_{i}=\frac{L\;R}{M\;p_{i}}\text{exp}\left(-{\left(\frac{a\;\Gamma_{i}}{\gamma_{i}}\right)}^{b}\right)\frac{\text{bits}}{\text{joule}}$$ (17)

**Fig 3:**
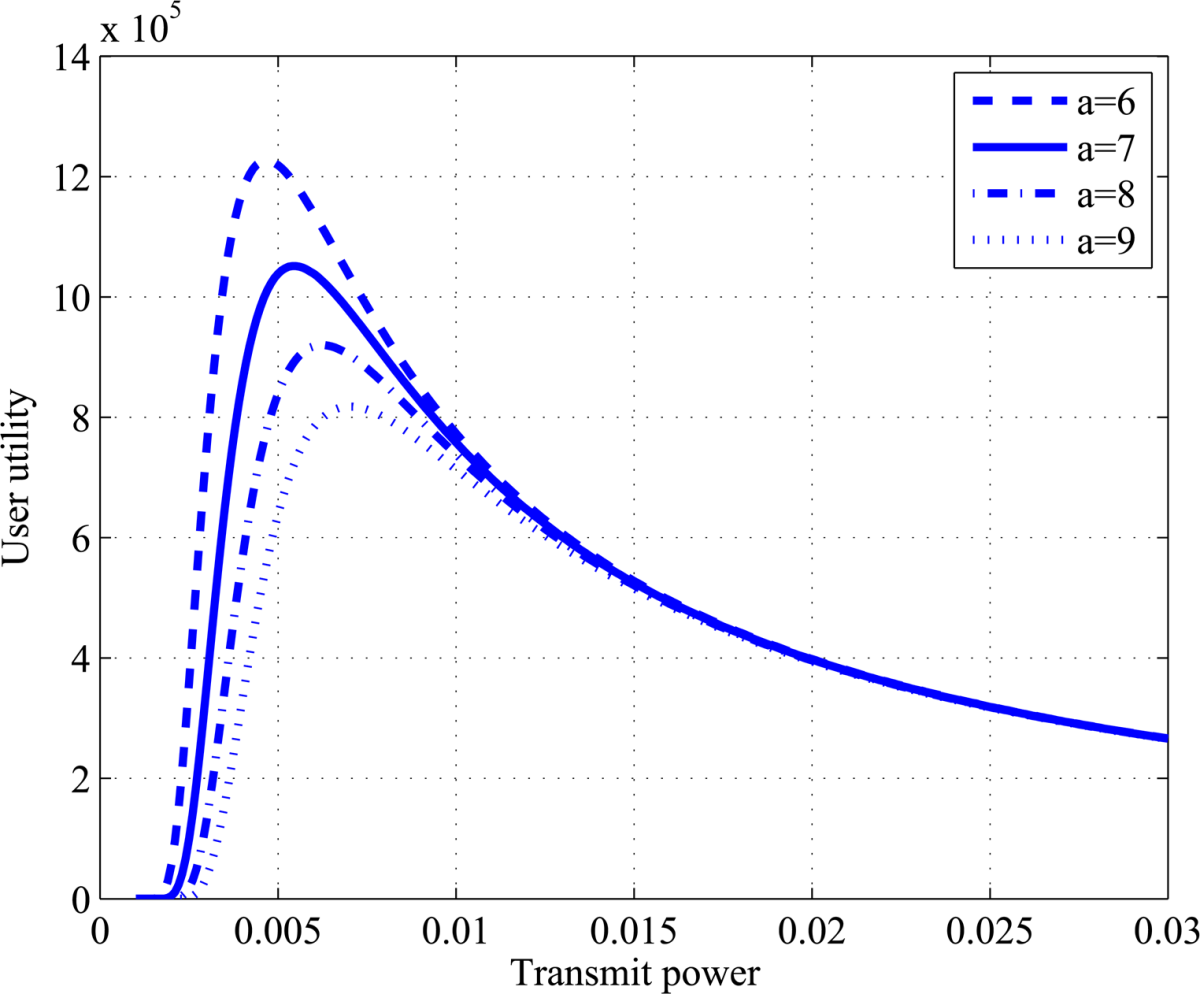
User’s utility function as a function of transmit power for fixed interference and different value of weighting factor *a*.

The utility function in Eq (17) represents the tradeoff between the throughput and battery life and it is particularly appropriate for applications where saving power is more important than achieving a high throughput, such as green cognitive radio [23]. Assuming that the value of target SIR is fixed at the cognitive radio system, the proposed utility function can be tuned using the weighting factor *a*. The user’s optimal transmit power will be changed depending on the maxima of utility function. Fig 3 shows the curves of our proposed utility function with respect to transmit power in different values of *a*. It is shown that the utility increases and the transmitting power decreases by decreasing the value of the parameter *a*, but this will decrease the target of SIR of the system. The factor *a* can be broadcast by the primary system to the cognitive networks to adjust the target SIR depending on the amount of interference. The primary system sends a lower value of *a* when the amount of interference approximately reaches the interference temperature limit.

**Fig 4:**
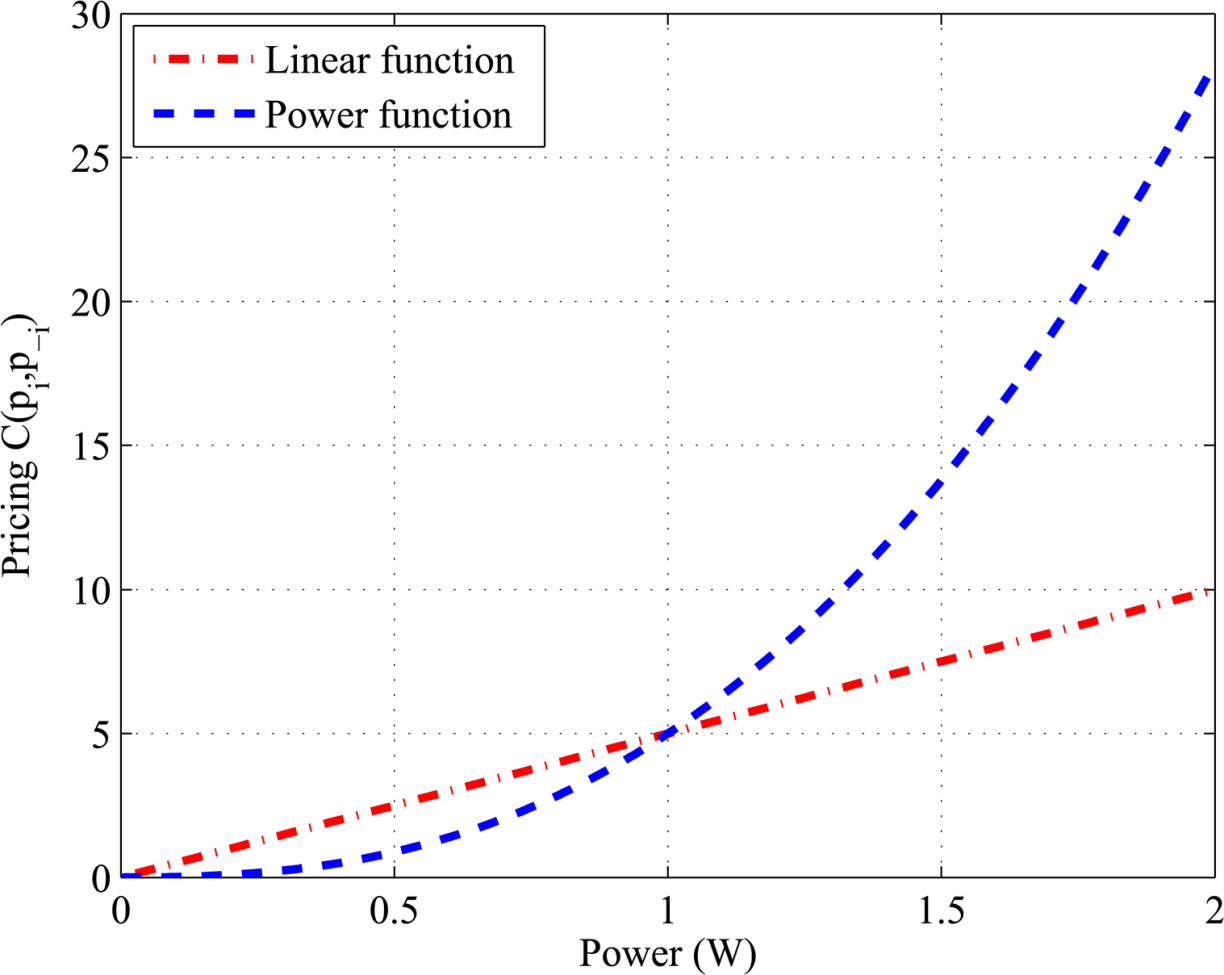
Linear and power function pricing comparison.

Further, we introduce a new design of the pricing function to improve the system performance by encouraging CRs to use system resources efficiently. The contribution in our design is to apply a high cost to the users that use high power, such as the farthest users from the base station. Therefore, we introduce a power function of the transmit power instead of traditional linear pricing. Fig 4 shows an example of the difference between the linear and power pricing techniques. We assumed that user transmit power varies between the minimum and maximum power strategy space [0, 2], and price functions computed numerically. It is shown that the power function pricing is lower than the linear function pricing for CRs who use low transmit power (closer users), whereas a high pricing cost will be applied to the CRs who use high transmit power (farthest users).

Thus, the proposed pricing function is expressed as

$$C_{i}(p_{i},p_{-\text{i}})=c\;p_{i}^{\alpha }$$ (18)

where *c* and *α* are the pricing factors. Thus, the utility function with pricing can be expressed as

$$U_{i}^{C}\;(p_{i},p_{-\text{i}})=\frac{L\;R}{M\;p_{i}}\text{exp}\left(-{\left(\frac{a\;\Gamma_{i}}{\gamma_{i}}\right)}^{b}\right)-c\;p_{i}^{\alpha }$$ (19)

Therefore, the proposed energy efficient non-cooperative power control in the game with pricing (EF-NPGP) is expressed as

$$\text{EF}-\text{NPGP}\;:\;\underset{p_{i}\in P_{i}}{\text{max}}\;U_{i}^{C}\;(p_{i},p_{-\text{i}})=\frac{L\;R}{M\;p_{i}}\text{exp}\left(-{\left(\frac{a\;\Gamma_{i}}{\gamma_{i}}\right)}^{b}\right)-c\;p_{i}^{\alpha }$$ (20)

The advantage of this pricing function is its ability to guide CRs to an efficient Nash Equilibrium point. This is done by increasing the cost of the farthest users who use high transmit power in their communication. Moreover, the pricing function reduced the cost applied to the nearest CRs who use low transmit power in their communication. Each CR seeks to maximize its own profit (utility-price) by adjusting its transmit power in a distributed manner. The expected Nash Equilibrium result from the power control game is the balanced power of all CRs that no single CR can increase the benefit by changing its own transmits power. To derive an algorithm of non-cooperative power control game, we adopt a power control algorithm in which each CR will maximize its net utility $U_{i}^{C}\;(p_{i},p_{-\text{i}})$. For power optimization, the maximization can be achieved at a point for which the partial derivative of $U_{i}^{C}\;(p_{i},p_{-\text{i}})$ with respect to power *p*_*i*_ is equal to zero. For simple analysis, we get

$$\frac{\partial U_{i}^{C}}{\partial p_{i}}=\frac{L\;R}{M\;p_{i}^{2}}\left(\gamma_{i}\frac{\partial f_{4}(\gamma_{i})}{\partial \gamma_{i}}-f_{4}(\gamma_{i})\right)-\alpha \;c\;p_{i}^{\alpha -1}$$ (21)

### Existence of Nash Equilibrium

In non-cooperative power control game, the *i*th CR maximizes its utility by choosing a proper strategy from the strategy set $P_{i}=[0,P_{i}^{\text{max}}]$.

A Nash Equilibrium exists in non-cooperative power control game, if for all *i* = 1,2,.., *n* meet the following two conditions [24]:

1. The action set *P*_*i*_ is non-empty, convex, and compact subset of some Euclidean $R^{N}$.
2. The utility function $U_{i}^{C}\;(p_{i},p_{-\text{i}})$ is continuous in **p** and $\left(\partial^{2}U_{i}^{C}/\partial p_{i}\partial p_{j}\right)\geq 0\;\forall \;j\neq i\in N$.

The transmit power space strategy for each CR in our game is defined by the minimum and maximum powers, and the value of powers is between these values. Therefore, the first condition of action set *P*_*i*_ is satisfied.

To show that the CR utility function is quasi-concave in *p*_*i*_, the second derivative of $U_{i}^{C}\;(p_{i},p_{-\text{i}})$ is obtained with respect to *p*_*i*_.

$$\frac{\partial U_{i}^{C}}{\partial p_{i}}=\frac{L\;R}{M\;p_{i}^{2}}\left(\gamma_{i}\frac{\partial f_{4}(\gamma_{i})}{\partial \gamma_{i}}-f_{4}(\gamma_{i})\right)-\alpha \;c\;p_{i}^{\alpha -1}$$ (22)

$$\begin{array}{l} \frac{\partial^{2}U_{i}^{C}}{\partial p_{i}\partial p_{j}}=\frac{L\;R}{M\;p_{i}^{2}}\left(\frac{\partial \gamma_{i}}{\partial p_{j}}\frac{\partial f_{4}(\gamma_{i})}{\partial \gamma_{i}}+\gamma_{i}\frac{\partial \gamma_{i}}{\partial p_{j}}\frac{\partial^{2}f_{4}(\gamma_{i})}{\partial \gamma_{i}^{2}}-\frac{\partial \gamma_{i}}{\partial p_{j}}\frac{\partial f_{4}(\gamma_{i})}{\partial \gamma_{i}}\right)\\ \;=\frac{L\;R}{M\;p_{i}^{2}}\left(\gamma_{i}\frac{\partial \gamma_{i}}{\partial p_{j}}\frac{\partial^{2}f_{4}(\gamma_{i})}{\partial \gamma_{i}^{2}}\right) \end{array}$$ (23)

Because the first-order derivative of *γ*_*i*_ with respect to *p*_*j*_ is $\left(\partial \gamma_{i}/\partial p_{j}\right)=-\left(Gh_{i}h_{j}p_{i}/\sum_{j\neq i}h_{j}p_{j}+\sigma^{2}\right)\;<\;0$, so we need the second-order derivative of our efficiency function with respect to *γ*_*i*_ be $\partial^{2}f_{4}(\gamma_{i})/\partial \gamma_{i}^{2}\leq 0$.

$$\begin{array}{l} \frac{\partial f_{4}(\gamma_{i})}{\partial \gamma_{i}}=\frac{\partial e^{\left(-{\left(\frac{a\;\Gamma_{i}}{\gamma_{i}}\right)}^{b}\right)}}{\partial \gamma_{i}}\\ \;=\frac{b\left(-{\left(\frac{a\;\Gamma_{i}}{\gamma_{i}}\right)}^{b}\right)e^{\left(-{\left(\frac{a\;\Gamma_{i}}{\gamma_{i}}\right)}^{b}\right)}}{\gamma_{i}} \end{array}$$ (24)

$$\frac{\partial^{2}f_{4}(\gamma_{i})}{\partial \gamma_{i}^{2}}=-\frac{b\;e^{\left(-{\left(\frac{a\;\Gamma_{i}}{\gamma_{i}}\right)}^{b}\right)}\left(b{\left(\frac{a\;\Gamma_{i}}{\gamma_{i}}\right)}^{b}+{\left(\frac{a\;\Gamma_{i}}{\gamma_{i}}\right)}^{b}-b{\left(\frac{a\Gamma_{i}}{\gamma_{i}}\right)}^{2b}\right)}{\gamma_{i}^{2}}$$ (25)

Because $b\;e^{\left(-{\left(\frac{a\;\Gamma_{i}}{\gamma_{i}}\right)}^{b}\right)}/\gamma_{i}\;>0$, in order to $\partial^{2}f_{4}(\gamma_{i})/\partial \gamma_{i}^{2}\leq 0$

$$\begin{array}{c} \left(b{\left(\frac{a\;\Gamma_{i}}{\gamma_{i}}\right)}^{b}+{\left(\frac{a\;\Gamma_{i}}{\gamma_{i}}\right)}^{b}-b{\left(\frac{a\;\Gamma_{i}}{\gamma_{i}}\right)}^{2b}\right)\;\geq 0\\ \Rightarrow b+1\geq \;b{\left(\frac{a\;\Gamma_{i}}{\gamma_{i}}\right)}^{b}\\ \frac{{\left(b+1\right)}^{1/b}}{a\;b}\geq \frac{\Gamma_{i}}{\gamma_{i}} \end{array}$$ (26)

According to Eq (26) and by selecting the pricing factors carefully, the second condition has been satisfied. Hence, the proposed power control game has a unique Nash Equilibrium solution.

### EF-NPGP algorithm

We suppose that each CR *i* updates its transmit power at time instances *T*_*i*_ = {*t*_*i*1_, *t*_*i*2_,….,} where *t*_*ik*_ < *t*_*i*(*k*+1)_ and by considering the proposed EF-NPGP as given in Eq (20), generate a sequence of powers as follows

**Fig 5:**
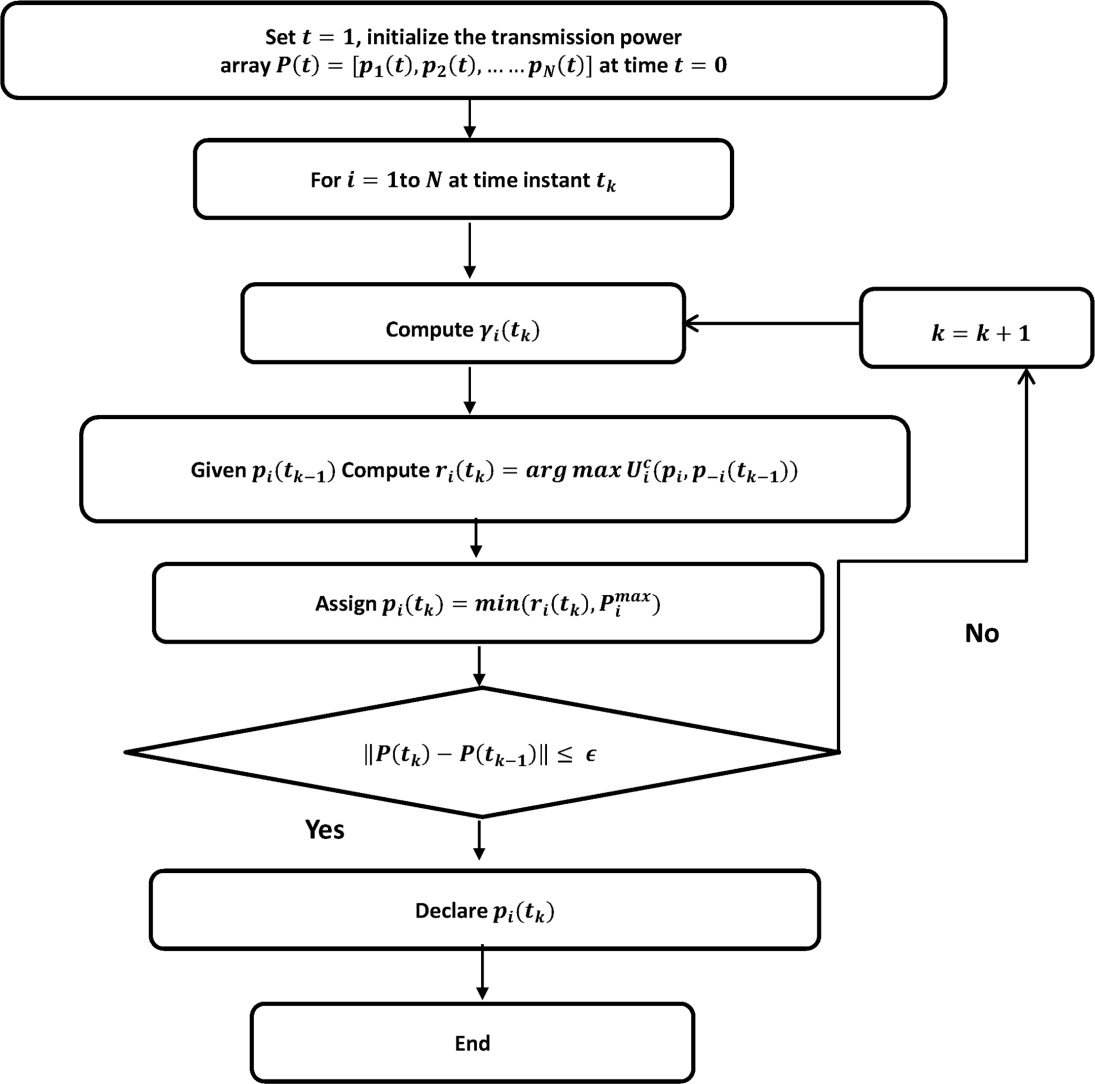
Flowchart illustrating the EF-NPGP algorithm.

- Initialize transmit power vector $p=[p_{1}^{0},p_{2}^{0},p_{3}^{0},\ldots ..,p_{N}^{0}]$ randomly at time *t*_0_
- For all *i* ∈ *N*, at time instant *t*_*k*_
  - Update *γ*_*i*_(*t*_*k*_) using Eq (1)
  - Given *p*_*i*_(*t*_*k*−1_), compute $r_{i}(t_{k})=\text{arg}\;\underset{p_{i}\in P_{i}}{\text{max}}\;U_{i}^{C}\;(p_{i},p_{-\text{i}}(t_{k-1}))$
  - Assign the transmit power as $p_{i}(t_{k})=\text{min}(r_{i}(t_{k}),\;p_{i}^{\text{max}})$
- If ‖*p*(*t*_*k*_) − *p*(*t*_*k*−1_)‖ ≤ *ε*, stop iteration and declare Nash Equilibrium as *p*(*t*_*k*_). Else, *k* = *k* + 1 and go to Step II.

where *r*_*i*_(*t*_*k*_) represents the set of best transmit powers for *i*th CR at time instant *k*. The flow chart of the proposed EF-NPGP algorithm is shown in Fig 5.

## Numerical Results and Discussion

In this section, we compare the performance of our proposed power control game algorithm with NPG_MSFLIA [17], NPG-ESIA [18], and R-NPGP [19]. The utility functions that have been used in the comparison are explained in Eqs (10), (12), and (14). We applied the same numerical computation to obtain the Nash Equilibrium solution of utility functions in order to present the advantages of our proposed utility function. The constant system parameters used in the simulation are listed in Table 2.

**Table 2:** System parameters.

Parameter	Value
Total number of bits per frame, *M*	80
Number of information bits of each frame, *L*	64
Spread spectrum processing gain, *G*	100
Data rate, *R*	10 kbps
AWGN power at receiver, *σ* ^2^	5e-15 Watts
Maximum power constraint, $p_{i}^{\text{max}}$	2 Watts
Target SIR, Γ_*i*_	10
Weighting factors *a*,*b*,*c*, and *α*	0.88, 3, 1e4, 2.5

We have considered a simple system model based on a single-cell cognitive radio CDMA system with a fixed packet size and no coding for forward error correction. For the general efficiency function defined in Eq (11), the equilibrium SIR is found by solving the formula *f*′(*γ*)*γ* – *f*(*γ*) = 0 that guarantee maximum utility of γ* = 12.4. The value of γ* is the real target SIR that all CRs achieve to maximize their own utility function. For the cognitive radio CDMA system, the feasibility condition for γ* is giving by the following bound on the number of users [25]:

$$N\leq 1+(G/\gamma^{*})=9.05\;\text{CR terminals}$$ (27)

According to (27), we assume that there are not more than 9 CRs in the system and their distance from the base station *d* = [368m, 490m, 580m, 630m, 720m, 810m, 950m, 1070m, 1140m].

In this paper, we use a simple propagation model in which all the path gains are deterministic functions, with path loss exponent *β*, of the distance between the cognitive radio *i* and CBS

$$h_{i}=\frac{K}{r_{i}^{\beta }}$$ (28)

where *r*_*i*_ is the distance between the *i*th user and base station, *β* is the path loss exponent, which is supposed to be 4 that is usually between 2 and 6, and *K* = 0.097 is a constant. This value of *K* = 0.097 is selected to establish a transmit power of 2 W for a CR terminal operating at 1140 meters from CBC in the system with 9 CRs, and all operating with γ*. In this simulation, all cognitive users start with initial power $p_{i}^{\left(0\right)}=2.22\times 10^{-16}\;\text{W}$ for all algorithms and *ε* = 10^−5^. In this simulation, the weighting and pricing factors have been tuned until all algorithms achieve the same average value of SIR.

**Fig 6:**
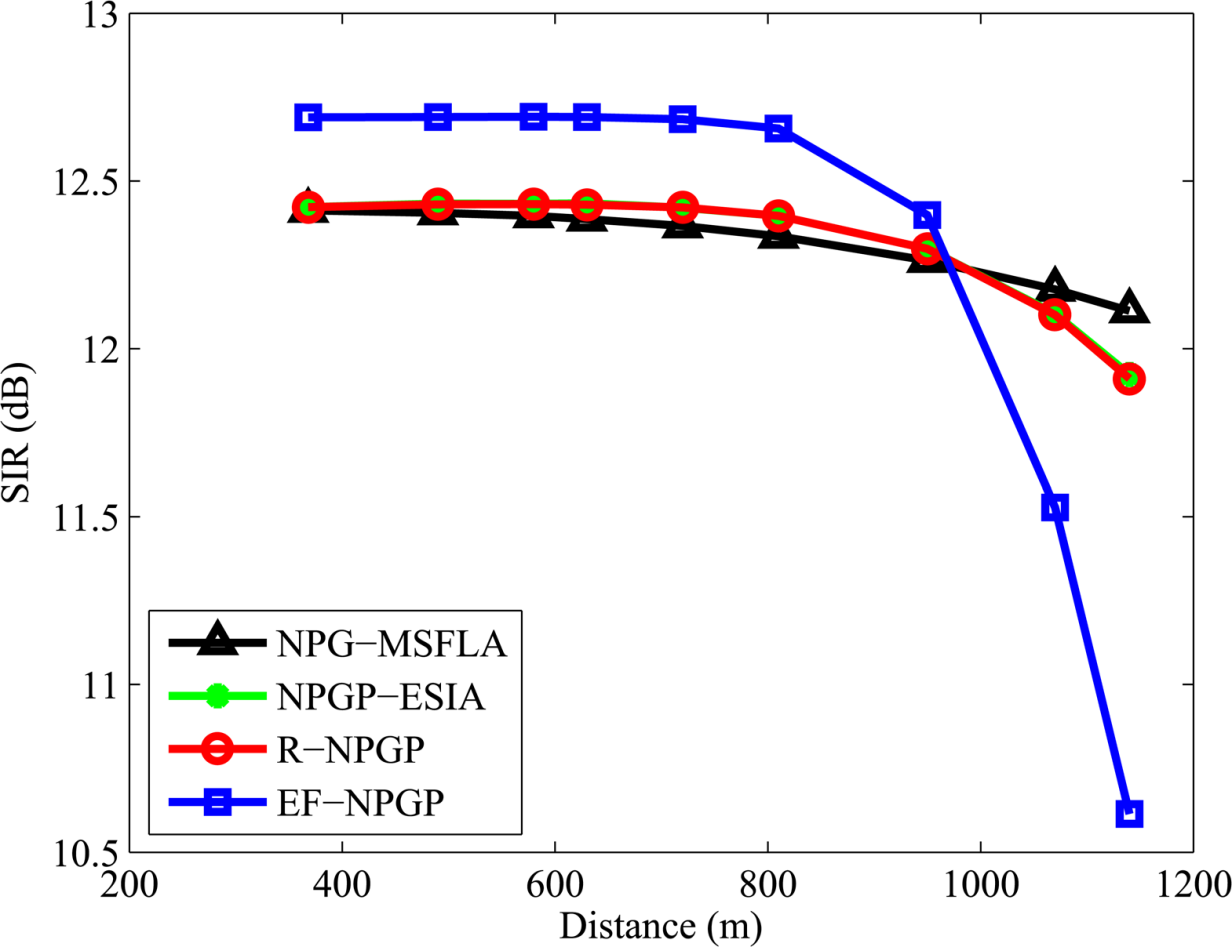
Comparison curves of each CR’s SIR for all algorithms.

**Fig 7:**
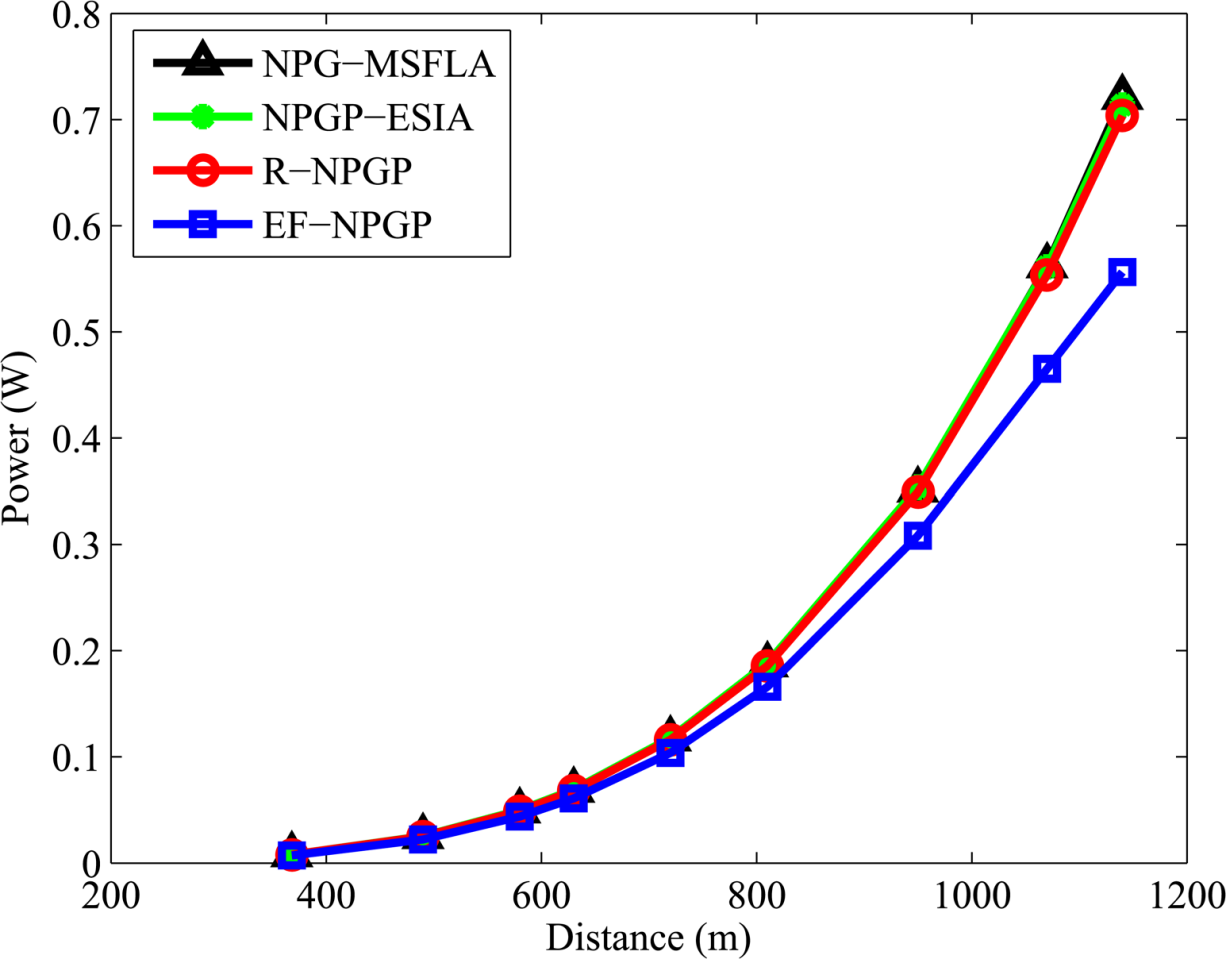
Comparison curves of each CR’s transmit power for all algorithms.

Fig 6 explains the results of SIR at Nash Equilibrium that are achieved by CR according to the distance between each CR and base station. All CR users maintain their SIR above the target value (Γ_*i*_ = 10) and SIR value is decreased by increasing the distance for all algorithms. The comparison of SIR for all algorithms shows that our proposed algorithm EF-NPGP is more efficient, especially for the first 7 users, where the values of SIRs are the highest comparing with other algorithms. The farthest CRs consume the highest powers and they represent the origin of interference, therefore our proposed algorithm applied a higher cost to the farthest users. Fig 7 shows the curves of transmit power in Watts with the distance between the CR and the base station for all algorithms where the transmit power increases gradually by increasing the distance of the user. It can be seen that the transmit power curve of the proposed EF-NPGP is the lowest comparing to NPG-MSFLIA, NPGP-ESIA, and R-NPGP.

Table 3 shows the SIR of CR users at the end of the network algorithm simulation. This table shows that the EF-NPGP with our proposed price function achieve the highest value of SIR. The SIR of the last two CR users is smaller due to applied higher cost. Therefore, it can reach a better equilibrium point by restricting the minimum required SIR for terminals with bad channel conditions.

**Table 3:** Final SIR of CR users.

CR user #	Final SIR of NPG_MSFLIA [17]	Final SIR of NPG-ESIA [18]	Final SIR of R-NPGP [19]	Final SIR of Proposed EF-NPGP
**1**	12.41	12.42	12.42	12.69
**2**	12.4	12.43	12.43	12.69
**3**	12.4	12.43	12.43	12.69
**4**	12.39	12.43	12.43	12.69
**5**	12.37	12.42	12.42	12.68
**6**	12.33	12.4	12.4	12.66
**7**	12.26	12.3	12.3	12.4
**8**	12.18	12.1	12.1	11.53
**9**	12.11	11.92	11.91	10.61

**Fig 8:**
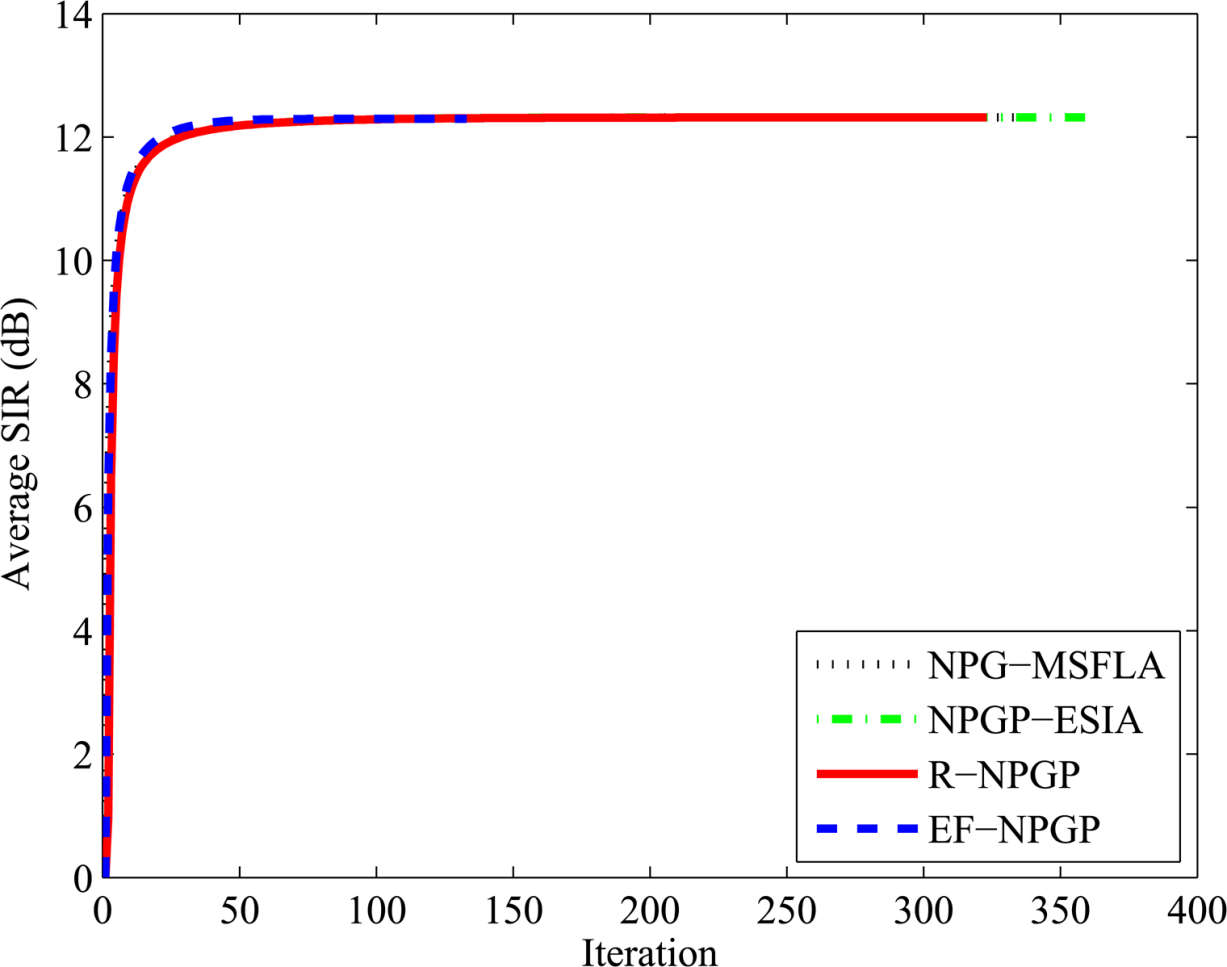
Comparison curves of average SIR for all algorithms with number of iterations.

**Fig 9:**
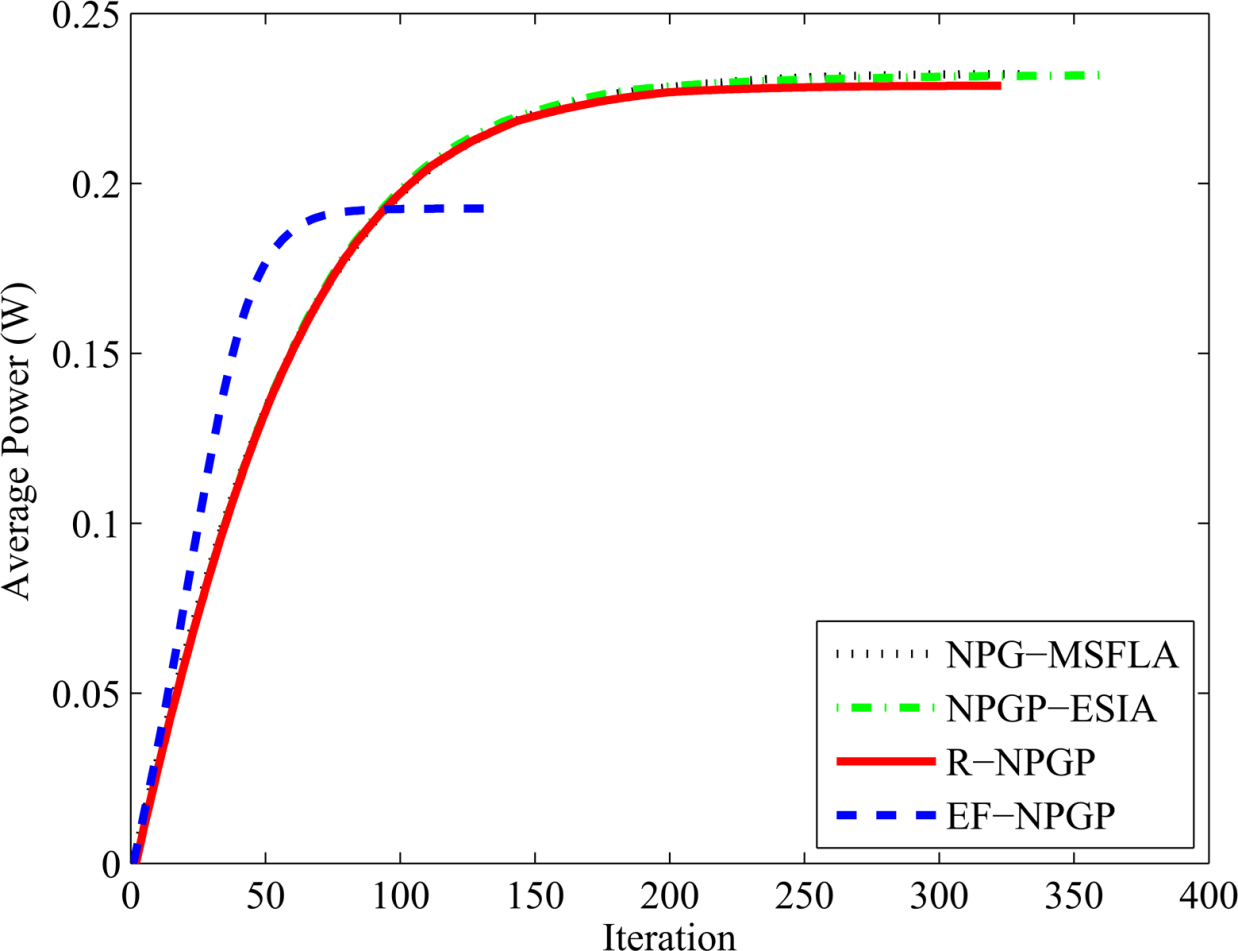
Comparison curves of average power for all algorithms with number of iterations.

Next, we test the average power and average SIR for all algorithms to determine the convergence speed of algorithms and the reduction of average power. In this test, the horizontal axis represents the iteration time that needs to obtain the Nash Equilibrium and the vertical axis represents the average SIR and average power. It is shown in Fig 8 that all algorithms approximately achieve the same value of average SIR without any significant differences, but the speeds of convergence are not equal. It is found that our proposed EF-NPGP algorithm can obtain the Nash Equilibrium with only 133 iterations while it needs 333, 360, and 323 for NPG-MSFLA, NPGP-ESIA and R-NPGP, respectively. On the other hand, Fig 9 shows the comparison curves of average transmit power obtained by all algorithms. It can be seen from Fig 9 that the average power consumption of the proposed EF-NPGP algorithm has a significant reduction comparing with other algorithms. The result obtained from Fig 9 indicates that the amount of interference measured at the primary system from the proposed EF-NPGP is the lowest comparing with other algorithms. This feature of the proposed EF-NPGP algorithm makes it the best for maximizing the spectrum sharing and QoS guarantees in both systems. The speed convergence of all algorithms can be seen clearly in Fig 9, in which it founds that our proposed EF-NPGP is the fastest comparing with other algorithms. In Table 4, we explained the average power in Watt, and the number of process iterations of all algorithms.

**Table 4:** Values of average power and number of iterations of algorithms.

Algorithm	Average Power (W)	Number of iterations
**NPG-MSFLA**	0.2321	333
**NPGP-ESIA**	0.2319	360
**R-NPGP**	0.2287	323
**EF-NPGP**	0.1926	133

**Fig 10:**
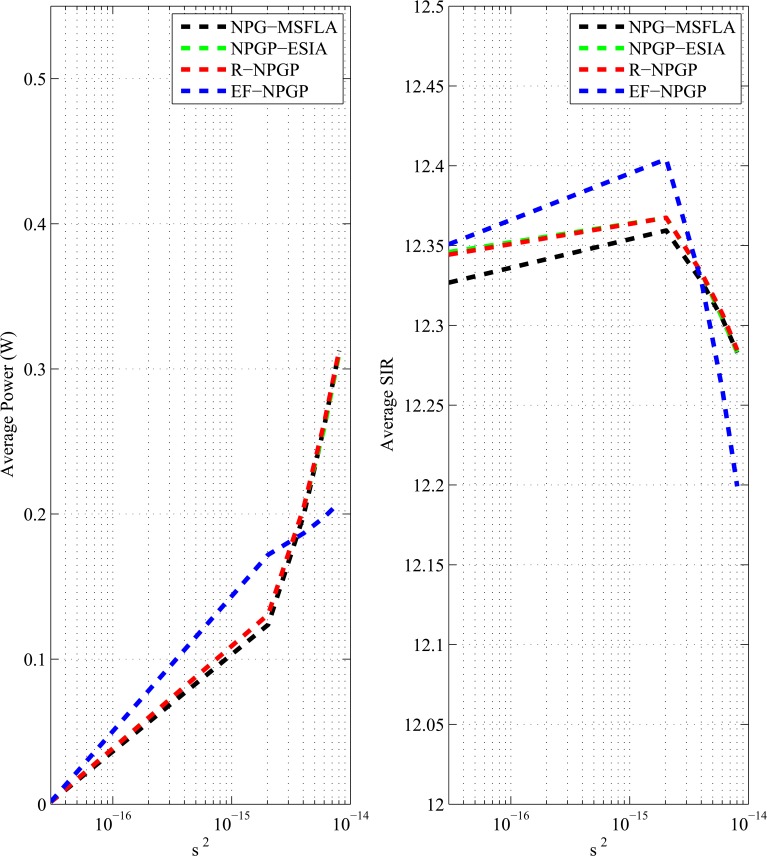
Performance comparison of average power and SIR for noise values.

The impact of noise to all algorithms is shown in Fig 10. We test the algorithm using the same parameters that applied to the previous test. We range the value of noise from 3 × 10^−17^ W to 10^−14^ W. As shown in Fig 10, the average power increases with the increase of noise because the power is proportional to noise, while the average SIRs decreased with the increase of noise because SIR is inversely proportional to noise as in Eq (1). The proposed EF-NPGP algorithm provides significant savings of power in high noise case, in which (*p*_*EF*−*NPGP*_ = 0.2078 W), and (*p* = 0.3127 W) for all other algorithms. On the other hand, the reduction in average SIR is insignificant compared to other algorithms.

## Limitations and Future Works

The main objective of this work is to reallocate the transmit power among CRs to maintain the required QoS of CRs to obtain a significant decrease in power consumption and mitigate the aggregate interference. Under no pricing, all above utilities are maximized at the same value of SIR, which can be obtained by solving the following formula

f′(γ)γ=f(γ) (29)

The value of SIR is determined according to the design of the efficiency function that depends on the system characteristic. As the pricing function is applied and the value of pricing factor is increased, CRs attain lower SIR, lower power, and higher utilities. Due to the pricing, SIRs are no longer equal at the equilibrium for all CRs. In actual fact, a user closer to the base station achieves higher SIR than a user farther away. The reduction of CR’s SIR, the increase of CR’s utilities, and the algorithm speed of convergence are depending on the selected values of pricing factors *c* and *α*. In future works, we will consider an addition algorithm to select the best values of pricing factors *c*_*BEST*_ and *α*_*BEST*_ that can be applied to the network (base station) side to get more significant improvement.

## Conclusion

We have presented a non-cooperative power control algorithm in cognitive radio networks. The QoS of a CR user refers to an efficient utility function via pricing. By introducing the new utility and price functions, an efficient non-cooperative power control game has been produced and the existence and uniqueness of Nash Equilibrium is proved. Numerical results indicate that the non-cooperative power control algorithm proposed in this paper has better power saving and faster convergence compared to recently available works in the literature. In addition, most of closer CR users in our proposed algorithm can meet higher SIR than the users in other algorithms. The higher pricing only applied to the farthest users that represent a higher source of undesirable interference. The proposed scheme offers an improved performance, in which the CRN can now share extra licensed band under the interference temperature limits. The significant reduction in the transmit power of the proposed power control algorithm gives the highest preference to apply it in cognitive radio sensor networks and green cognitive radio networks. As future work, it is suggested to study the proposed algorithm with the higher load system and with the existence of primary users.
